# Gut microbiota dysbiosis and protein energy wasting in patients on hemodialysis: an observational longitudinal study

**DOI:** 10.3389/fnut.2023.1270690

**Published:** 2024-01-10

**Authors:** Xiao Bi, Yuqing Liu, Lu Yao, Lilu Ling, Jianxin Lu, Chun Hu, Wei Ding

**Affiliations:** Division of Nephrology, Shanghai Ninth People’s Hospital, School of Medicine, Shanghai Jiaotong University, Shanghai, China

**Keywords:** hemodialysis, protein-energy wasting, gut microbiota, skeletal muscle mass, *Bifidobacterium*

## Abstract

**Background:**

Protein energy wasting (PEW) is common in patients on hemodialysis, and its development may involve gut microbial dysbiosis. However, the exact relationship between the composition of different flora and the development of PEW remains unclear.

**Methods:**

This is an observational longitudinal study on 115 patients undergoing hemodialysis who were followed up for 1 year. All the patients were evaluated at baseline, and different microbiota compositions were determined. After a 1 year follow-up period, the correlations between clinical parameter variations and the relative abundance of different gut flora were assessed using Spearman correlation. Moreover, the associations of the abundance of different gut microbiota with decrease in lean tissue mass and the development of PEW were analyzed using ROC curve and logistical regression analyses.

**Results:**

We found that the relative abundances of Actinobacteria and Bifidobacteriaceae were significantly lower in patients with PEW than in those who did not develop PEW (*p* < 0.05). The abundance of Actinobacteria and Bifidobacteriaceae correlated positively with variations in serum albumin levels (*r* = 0.213, *p* = 0.035 and *r* = 0.214, *p* = 0.034, respectively), lean tissue mass (*r* = 0.296, *p* = 0.007 and *r* = 0.238, *p* = 0.002, respectively), and lean tissue index (*r* = 0.377, *p* < 0.001 and *r* = 0.419, *p* < 0.001, respectively). The area under the ROC curve or AUC values of Actinobacteria and Bifidobacteriaceae for the prediction of lean tissue mass decrease ranged from 0.676 to 0.708 (*p* < 0.05). Thus, decrease in the abundance of Actinobacteria and Bifidobacteriaceae may be associated with decrease in lean tissue mass and the occurrence of PEW.

**Conclusion:**

The present findings imply Actinobacteria and Bifidobacteriaceae may be potential markers for predicting skeletal muscle mass decrease and PEW development in patients on hemodialysis.

## 1 Introduction

Protein-energy wasting (PEW) commonly occurs in patients with end-stage renal disease (ESRD), especially in those who are on maintenance dialysis treatment (1). Its incidence is 30% to 75% among patients on hemodialysis (2), and it results in poor quality of life and survival rate in these patients (3). PEW is associated with adverse events, such as mortality and morbidity, especially in patients with chronic dialysis (4). It is characterized by a reduction in the body’s reserves of protein and energy, low serum albumin and pre-albumin concentrations, reduced body mass index (BMI), and insufficient dietary protein intake. Several factors contribute to the development of PEW, including decrease in daily nutrient intake, inadequate dialysis, metabolic derangements, inflammation, and comorbid conditions (5). With regard to the mechanisms underlying PEW, there are lack of standard treatments against PEW. It is important to have a deeper understanding of the mechanisms associated with PEW in order to improve its treatment and prognosis.

The gut microbiota refers to the microorganisms that inhabit the gastrointestinal tract, and it is composed of bacteria, viruses, archaea, protists, and fungi. More than 100 trillion cells are present in the gastrointestinal tract. The dominant microbiota include Firmicutes, Bacteroidetes, Actinobacteria, and Proteobacteria, which are part of the normal flora that determines the physiological and pathophysiological features of the host (6, 7). With the assistance of advanced bioinformatics techniques, including the bacterial 16S ribosomal RNA (16S rRNA) gene sequencing, alterations in gut microbiota homeostasis have been detected in many human diseases, such as diabetes, chronic kidney disease, and irritable bowel disease (8, 9). In patients with chronic kidney disease (CKD), the gut microbial diversity is obviously reduced, and the microbial community differs significantly from that found in healthy individuals (10, 11). Further, metabolites and toxins produced by the gut microbiota are strongly linked with the progression of CKD and its complication cardiovascular disease (12). Recent reports have demonstrated that gut microbiota might be involved in the development of insulin resistance and metabolic acidosis, which play an important role in the pathogenesis of PEW (13, 14). Accordingly, fecal microbiota transplantation performed in healthy free-germ mice using cecal bacterial samples obtained from CKD mice was found to induce insulin resistance, systemic microinflammation, and sarcopenia (15). Further, two recent cross-sectional studies indicated intestinal flora disorders in patients on hemodialysis who developed PEW (16, 17). One study found that a butyrate-producing bacteria named *Faecalibacterium prausnitzii* was markedly reduced in PEW patients evaluated by SGA scores. The other study demonstrated positive correlations between butyric acid-producing bacteria (Rosella and Phascolarctobacterium) and PEW indicators such as grip strength and BMI. However, it is difficult to explain by cross-sectional studies whether and in which way disruption of intestinal flora contributes to the development of PEW in hemodialysis patients.

The present study sought to fill in the research gap discussed above by exploring the associations between gut microbiota disruption and the development of PEW through an observational longitudinal investigation into the gut microbial profile of fecal samples from patients on hemodialysis. Identification of the species that contribute to PEW development may help shed light on the mechanisms of PEW and develop treatments against uremic dysbiosis (18).

## 2 Methods

### 2.1 Study design and participants

**Figure 1:**
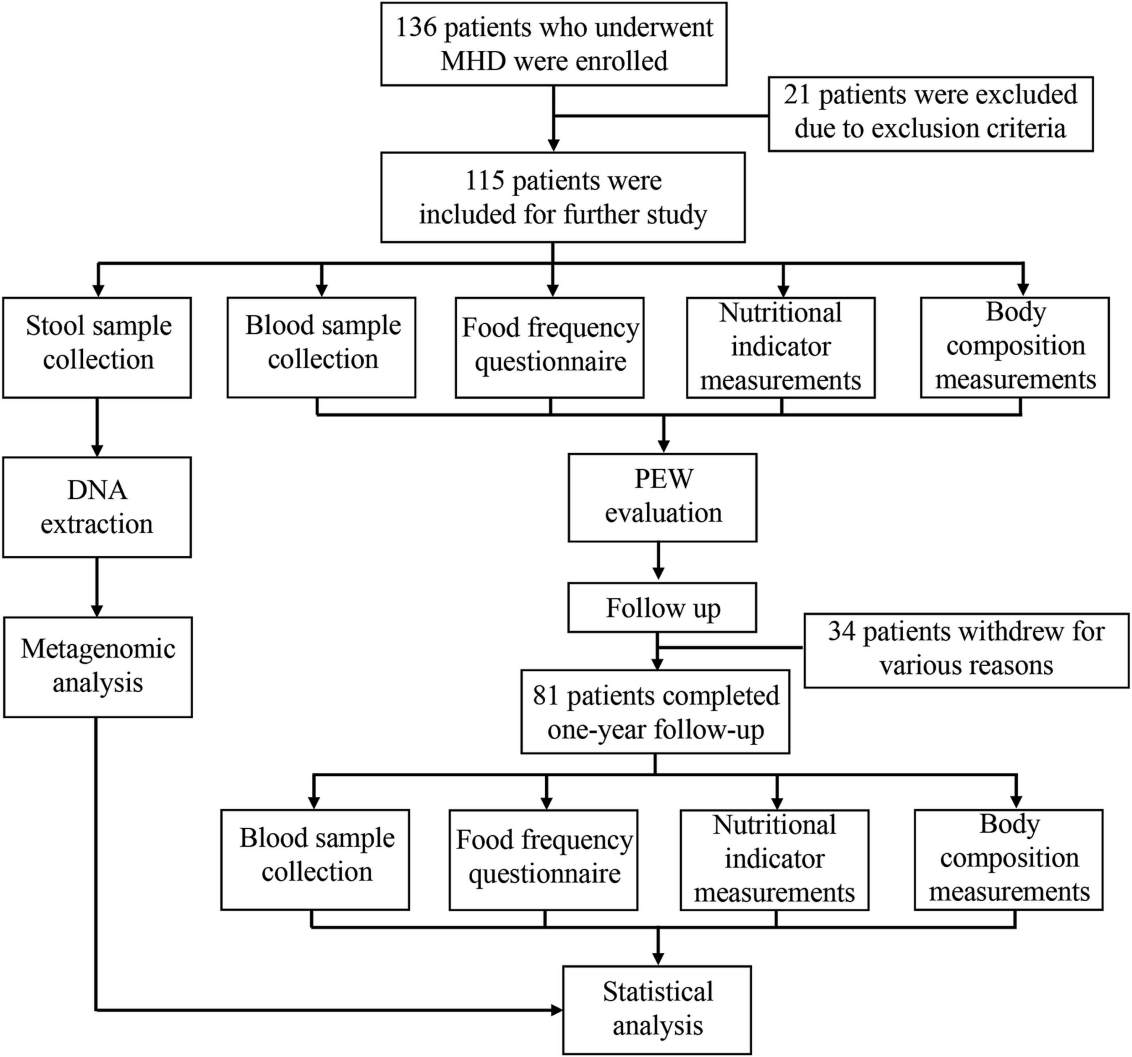
Flow chart depicting the research protocol.

This was an observational longitudinal study that enrolled 136 patients on maintenance hemodialysis from the Shanghai 9th People’s Hospital Hemodialysis Center. Among the 136 patients, 16 patients with a history of autoimmune and inflammatory diseases, heart failure, cancer, chronic liver disease, acute infection, and chronic diarrhea were excluded. Further, 5 patients who had used antibiotics during the 3 months period before screening for the study were also excluded. Finally, 115 patients were included in the study. All the patients had undergone hemodialysis three times a week for at least 3 months and were anuric, clinically stable, and free of obvious edema. Blood samples and fecal samples were collected at baseline. Blood samples were drawn before dialysis at the mid-week dialysis session and sent to our central laboratory for measurement. To evaluate the dietary protein intake (DPI) of each patient, the dietary intake of each patient was recorded over three consecutive days, including two weekdays and a half weekend. Measurements of body composition, as well as nutritional indicators such as skin fold, grip strength, and mid-arm circumference, were also performed at baseline. Patients were evaluated for PEW status as explained below, and they were followed up for 1 year. During the follow-up period, 20 patients withdrew from the study due to use of antibiotics or supplementation with probiotics. In addition, 8 patients died of various causes, 2 patients were transferred to other hemodialysis centers, and 4 patients withdrew for personal reasons. Therefore, only 81 patients completed the 1 year follow-up and were reevaluated for PEW status. The research flow chart is presented in Figure 1.

### 2.2 Assessment of PEW status

The indicators for diagnosis of PEW in this study were in accordance with the criteria proposed by the International Society of Renal Nutrition and Metabolism in 2008. The indicators were as follows: (a) serum albumin level <38 g/L, serum pre-albumin level <0.3 g/L, or serum cholesterol level <5.56 mmol/L; (b) BMI <23, unintentional weight loss >5% in the last 3 months, or unintentional weight loss >10% in the last 6 months; (c) >5% reduction in muscle mass over the last 3 months, >10% reduction in muscle mass over the last 6 months, or >10% reduction in mid-arm muscle circumference in relation to the 50th percentile value of the reference population; (d) unintentional DPI <0.8 g/kg per day.

### 2.3 Body composition measurements

Body composition variables were assessed using bioelectrical impedance analysis with a medical body composition analyzer (mBCA; Seca GmbH, Hamburg, Germany). The measurements were conducted according to the manufacturer’s instructions. Measurements were taken at 19 different frequencies ranging from 1 to 1,000 kHz. The mBCA provides measures of fat tissue index (FTI), lean tissue mass (LTM), total body water (TBW), extracellular water (ECW), and phase angel.

### 2.4 Fecal sample collection and DNA extraction

Fecal samples were freshly collected and stored at −80°C for subsequent analysis. Total genomic DNA from the samples was extracted according to the CTAB protocol. The V3–V4 region of the 16S ribosomal RNA gene was amplified with the specific barcode-indexed primers 341F and 805R. All the samples were sequenced using Illumina MiSeq 2000 at the same time in the same laboratory. On a per-sample basis, pair-end reads were merged using USEARCH (v8.0.1623), with 8 bp set as the minimum overlap of read pairs. Merged sequences were processed with the Mothur Software. Sequence analyses were performed with the Uparse software (version 7.0.1001) (Edgar, 2013), and sequences with ≥97% similarity were assigned to the same operational taxonomic units (OTUs). Following this, the taxonomic units were aligned based on the Greengenes reference database.

### 2.5 Bioinformatics analysis

Alpha diversity was calculated using the QIIME software to estimate the richness or evenness of taxa within each sample. Linear discriminant analysis (LDA) effect size (LEfSe) was employed to determine taxa that were significantly different between the PEW and non-PEW samples. The LDA value was set to 3. The results were plotted in a cladogram to depict the phylogenetic relationships between the identified taxa.

### 2.6 Statistical analysis

The data are presented as the mean ± standard deviation values or median and interquartile ranges, according to their distribution. Normally distributed data were analyzed using the student *t*-test, while non-normally distributed data were assessed by the Mann–Whitney *U*-test. The Fisher exact test was applied for comparison of categorical data. Linear correlations between relative abundances of microbial taxa and some clinical parameters were assessed by Spearman’s correlation analysis. Univariate and multivariate logistic regression models were developed to evaluate odds ratios and 95% confidence intervals for decrease in LTM or PEW in each microbial taxon. The predictive value of the microbial taxa was evaluated by calculating the area under the receiver operating characteristic curve (AUC). *p*-values <0.05 were regarded to indicate statistical significance. All statistical analyses were performed using Statistical Package for the Social Sciences software, version 27.0.

## 3 Results

### 3.1 Patient baseline characteristics

A total of 115 patients were included in this study. As shown in Table 1, 55 patients were diagnosed with PEW according to the diagnostic criteria for PEW. No significant differences were found between the PEW group and non-PEW group with regard to age, gender, triglyceride, hemoglobin, C-reactive protein, cholesterol, low-density lipoprotein, high-density lipoprotein, ferritin, uric acid, parathyroid hormone, skinfold thickness, and the level of inflammatory indicators (such as IL-6 and TNF-α) (*p* > 0.05). BMI, albumin concentration, pre-albumin concentration, and grip strength were significantly higher in the non-PEW group than in the PEW group (*p* < 0.05). LTM and LTI were lower in the PEW group than in the non-PEW, but the difference was not statistically significant (*p* = 0.059 for LTM; *p* = 0.055 for LTI).

**Table 1:** Characteristics of participants in the PEW and non-PEW groups.

	PEW	Non-PEW	*p*-value
Age (years)	62.8 ± 13.1	60.1 ± 14.1	NS
Gender (male: %, *n*)	60.0% (33/55)	65.0% (39/60)	NS
BMI (kg/m^2^)	22.1 ± 4.0	24.1 ± 5.4	0.023
Triglycerides (mmol/L)	3.9 ± 0.9	4.1 ± 1.0	NS
Hemoglobin (g/L)	107.7 ± 17.1	110.4 ± 13.3	NS
CRP (mg/L)	10.7 ± 3.7	5.8 ± 2.2	NS
Cholesterol (mmol/L)	1.9 ± 1.4	2.9 ± 2.4	0.011
LDL (mmol/L)	2.5 ± 0.8	2.5 ± 07	NS
HDL (mmol/L)	1.0 ± 0.3	1.0 ± 0.3	NS
Albumin (g/L)	37.2 ± 3.7	40.5 ± 2.2	<0.001
Pre-albumin (g/L)	0.33 ± 0.08	0.41 ± 0.06	<0.001
Ferritin (ng/mL)	279.3 ± 28.1	278.8 ± 42.5	NS
Uric acid (μmol/L)	413.2 ± 76.5	430.4 ± 83.9	NS
PTH (pmol/L)	305.1 ± 33.8	379.9 ± 41.2	NS
IL-6 (pg/mL)	19.1 ± 6.9	7.4 ± 1.4	0.084
TNF-α (pg/mL)	23.9 ± 22.7	19.7 ± 11.1	NS
ST (cm)	10.2 ± 4.9	10.9 ± 4.2	NS
MAC (cm)	25.9 ± 4.9	27.4 ± 3.7	0.06
Grip strength (kg)	20.3 ± 10.4	26.0 ± 10.5	0.005
DCI (kcal/kg/d)	25.5 ± 8.1	27.7 ± 7.0	NS
DPI (g/kg/d)	1.20 ± 0.21	1.16 ± 0.33	NS
Dialysis duration (y)	3.3 (1.8–11.2)	3.6 (2.1–7.5)	NS
Kt/v	1.4 (1.2–1.6)	1.4 (1.2–1.7)	NS
LTM (kg)	35.8 ± 9.1	39.5 ± 9.9	0.059
LTI (kg/ m^2^)	13.1 ± 2.6	14.2 ± 3.0	0.055

The mean ± SD values are presented for variables with normal distribution, while the median (IQR) values are presented for variables with abnormal distribution. BMI, body mass index; CRP, C-reactive protein; LDL, low-density lipoprotein; HDL, high-density lipoprotein; ST, skinfold thickness; MAC, mid-arm circumference; DCI, dietary calorie intake; DPI, dietary protein intake; LTM, lean tissue mass; LTI, lean tissue index.

### 3.2 Biodiversity of gut microbiota

**Figure 2:**
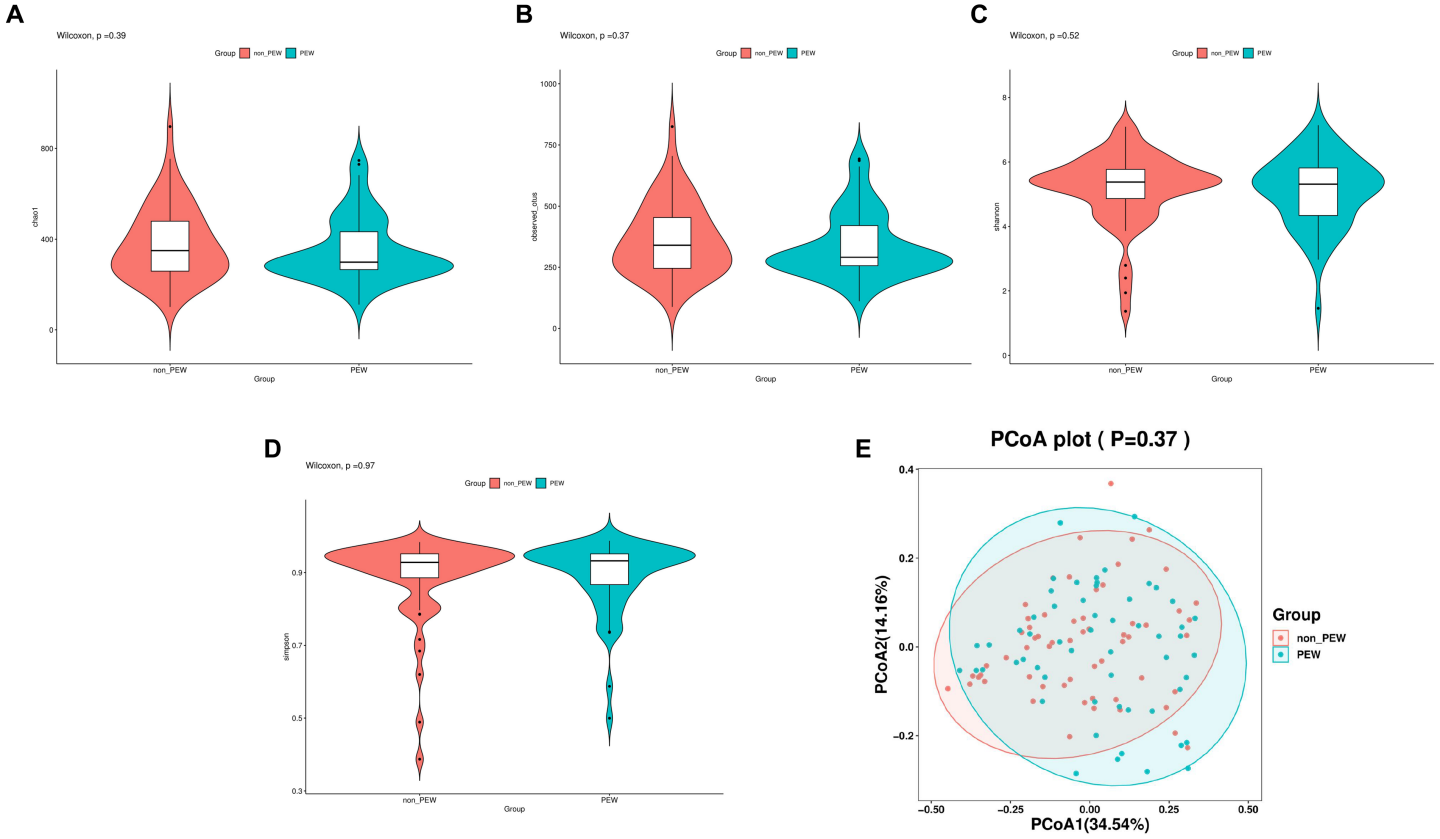
Comparison of different metrics of α-diversity and β-diversity between the PEW group and non-PEW group. **(A)** Chao1 index, **(B)** observed species index, **(C)** Shannon index, **(D)** Simpson index, **(E)** PCoA plot.

Alpha diversity was evaluated based on the Chao1, observed species, Shannon, and Simpson indexes. As shown in Figure 2, the alpha diversity indices were not significantly different between the two groups (*p* > 0.05). Further, beta diversity was also not significantly different between the two groups according to the results of principal co-ordinates analysis.

### 3.3 Differences in the composition of intestinal flora

**Figure 3:**
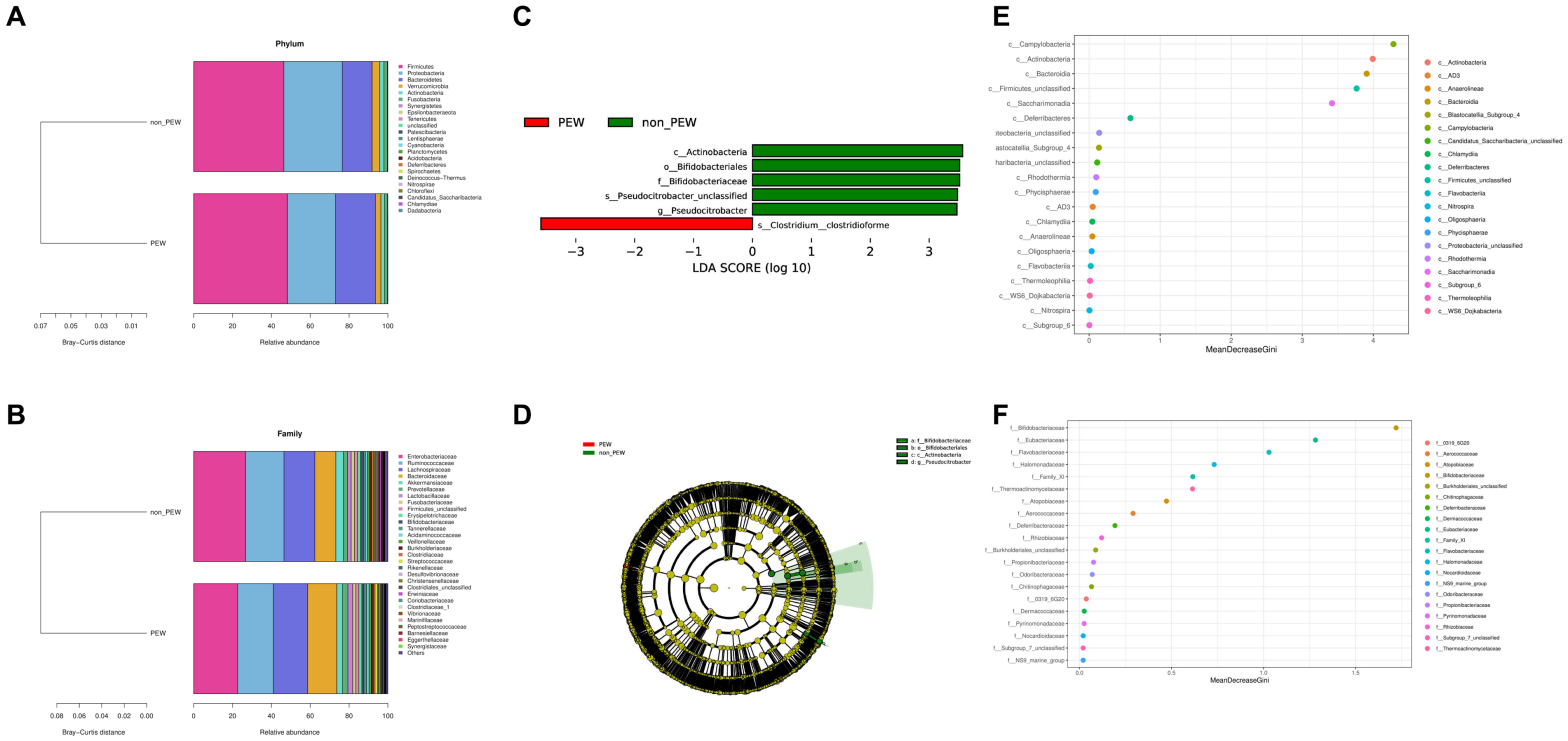
Fecal microbial comparison between the PEW group and non-PEW group. **(A)** Composition of gut microbiota at the phylum level; **(B)** composition of gut microbiota at the family level; **(C)** differential taxon features identified by LEfSe (LDA >3); **(D)** cladogram showing differentially abundant taxa of the gut microbiota; **(E,F)** differential taxon features assessed by random forest regression model at the class level **(E)** or family level **(F)**.

**Figure 4:**
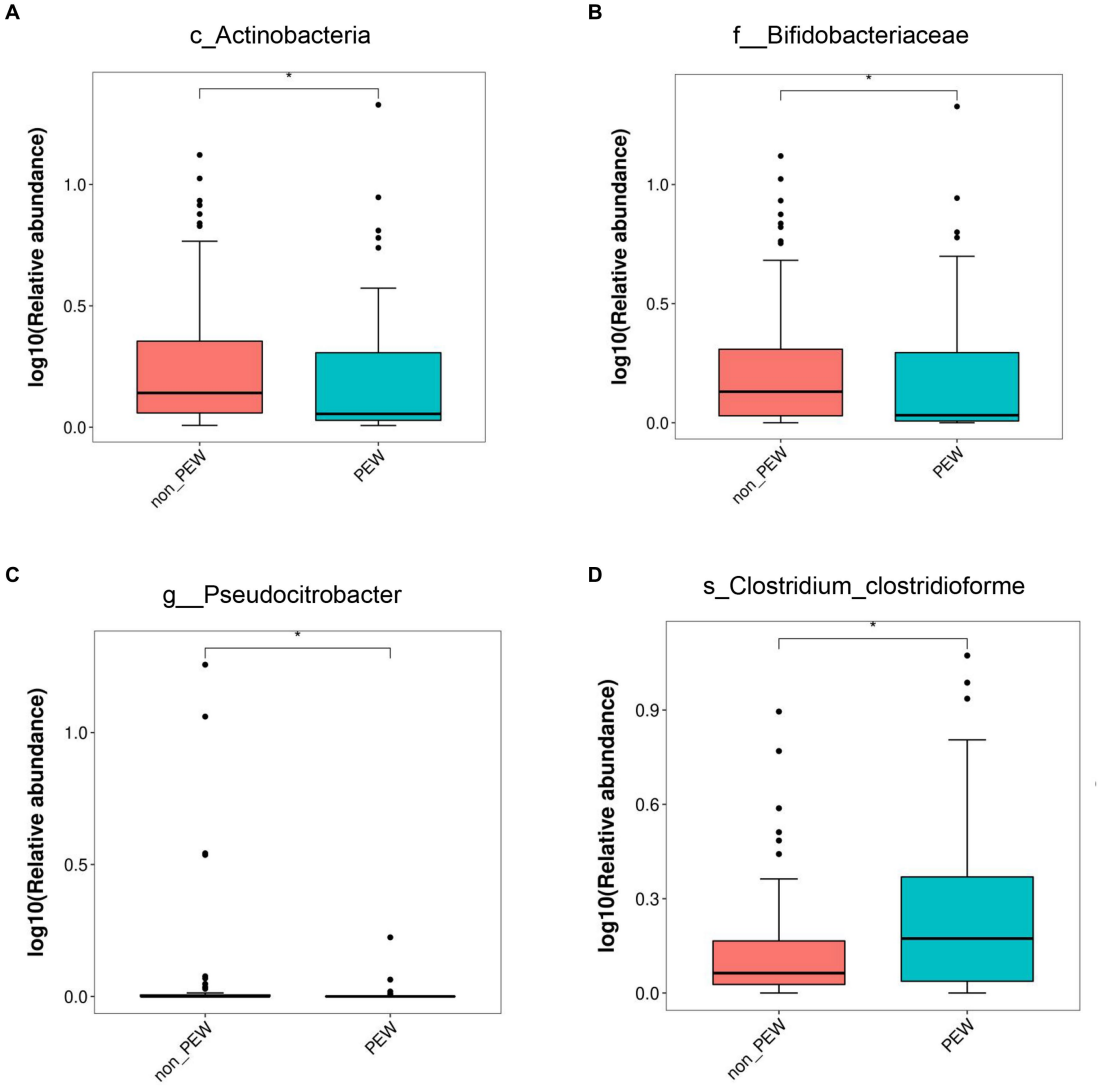
Relative abundance of gut microbiota in the PEW group and non-PEW group. The relative abundances of different taxa were compared between the two groups with the LEfSe method. **(A)** Actinobacteria at class level. **(B)** Bifidobacteriaceae at family level. **(C)** Pseudocitrobacter at genus level. **(D)** Clostridium_clostridioforme at species level. ^*^p < 0.05.

To determine the differences in microbiota composition between the PEW group and non-PEW group, we compared the microbiota at different levels. At the phylum level, the flora was mainly composed of Firmicutes, Proteobacteria, Bacteroidetes, Verrucomicrobia, and Actinobacteria (Figure 3A). At the family level, the flora was mainly composed of Enterobacteriaceae, Ruminococcaceae, Lachnospiraceae, Bacteroidaceae, and Akkermansiaceae (Figure 3B). The LEfSe method was used to identify microbial taxa that could be used to discriminate between different groups, with LDA >3 and *p* < 0.05 considered to indicate significant differences. As shown in Figures 3C,D, Actinobacteria at the class level, Bifidobacteriales at the order level, and Bifidobacteriaceae at the family level were significantly enriched in the non-PEW group compared with the PEW group. Furthermore, *Pseudocitrobacter* at the genus level and *Clostridium clostridioforme* at the species level also showed significant difference in abundances between the two groups. By applying the random forest regression model, we also found that either Actinobacteria at the class level or Bifidobacteriaceae at the family level was one of the most significant features that distinguished the PEW group from the non-PEW group (Figures 3E,F). The relative abundances of the identified microbial taxa between the two groups are depicted in Figure 4.

### 3.4 Correlations between different gut microbiota and variations in clinical parameters

To explore the potential role of different gut microbiota, we assessed the linear correlations between the relative abundance of the identified microbial taxa and variations in ST, MAC, LTM, LTI, BMI, DPI, and some other clinical parameters by Spearman correlation analysis. The variations were presented as the ratio of the value after 1 year of follow-up to the value at baseline. As shown in Table 2, the relative abundance of Actinobacteria at the class level correlated positively with variations in albumin (*r* = 0.213, *p* = 0.035), LTI (*r* = 0.377, *p* < 0.001), and LTM (*r* = 0.296, *p* = 0.007). Similarly, the relative abundance of Bifidobacteriaceae at the family level also correlated positively with variations in albumin (*r* = 0.214, *p* = 0.034), LTI (*r* = 0.419, p < 0.001), and LTM (*r* = 0.238, *p* = 0.002). Further, the relative abundance of *C. clostridioforme* at the species level correlated positively with variations in albumin (*r* = 0.279, *p* = 0.005).

**Table 2:** Linear correlations between relative abundances of different gut flora and variations in some of the clinical parameters.

	c__Actinobacteria	f__Bifidobacteriaceae	g__Pseudocitrobacter	s__Clostridium_clostridioforme
Hemoglobin (g/L)	*r* = −0.080 *p* = 0.43	*r* = −0.080 *p* = 0.427	*r* = −0.005 *p* = 0.964	*r* = 0.043 *p* = 0.674
CRP (mg/L)	*r* = 0.009 *p* = 0.934	*r* = 0.017 *p* = 0.867	*r* = 0.166 *p* = 0.104	*r* = −0.060 *p* = 0.556
Albumin (g/L)	***r* = 0.213** ***p* = 0.035**	***r* = 0.214** ***p* = 0.034**	*r* = 0.061 *p* = 0.552	***r* = 0.279** ***p* = 0.005**
Prealbumin (g/L)	*r* = 0.060 *p* = 0.555	*r* = 0.116 *p* = 0.253	*r* = 0.171 *p* = 0.092	*r* = 0.044 *p* = 0.669
Uric acid (μmol/L)	r = 0.087 *p* = 0.394	r = 0.053 *p* = 0.606	r = −0.094 *p* = 0.356	r = 0.182 *p* = 0.074
Triglyceride (mmol/L)	*r* = −0.155 *p* = 0.266	*r* = −0.111 *p* = 0.282	*r* = 0.034 *p* = 0.743	*r* = −0.032 *p* = 0.755
Cholesterol (mmol/L)	*r* = 0.053 *p* = 0.605	*r* = 0.053 *p* = 0.609	*r* = 0.007 *p* = 0.944	*r* = 0.050 *p* = 0.626
VitD3 (ng/mL)	*r* = 0.116 *p* = 0.272	*r* = 0.122 *p* = 0.251	*r* = 0.120 *p* = 0.256	*r* = −0.001 *p* = 0.991
PTH (ρmol/L)	*r* = −0.052 *p* = 0.613	*r* = −0.058 *p* = 0.574	*r* = 0.028 *p* = 0.723	*r* = 0.038 *p* = 0.715
Ferritin (ng/mL)	*r* = 0.044 *p* = 0.675	*r* = 0.039 *p* = 0.711	*r* = 0.097 *p* = 0.349	*r* = 0.092 *p* = 0.374
IL-6 (ρg/mL)	*r* = 0.020 *p* = 0.854	*r* = 0.070 *p* = 0.526	*r* = −0.068 *p* = 0.539	*r* = −0.060 *p* = 0.584
TNF-α (ρg/mL)	*r* = 0.030 *p* = 0.783	*r* = 0.064 *p* = 0.560	*r* = 0.023 *p* = 0.833	*r* = −0.015 *p* = 0.892
BMI (kg/m^2^)	*r* = 0.041 *p* = 0.694	*r* = 0.049 *p* = 0.639	*r* = 0.043 *p* = 0.682	*r* = 0.051 p = 0.626
ST (cm)	*r* = 0.163 *p* = 0.114	*r* = 0.158 *p* = 0.125	*r* = −0.109 *p* = 0.292	*r* = 0.050 *p* = 0.630
MAC (cm)	*r* = 0.135 *p* = 0.190	*r* = 0.080 *p* = 0.437	*r* = 0.037 *p* = 0.717	*r* = 0.062 *p* = 0.547
Grip strength (kg)	*r* = −0.132 *p* = 0.202	*r* = −0.118 *p* = 0.253	*r* = −0.003 *p* = 0.975	*r* = 0.086 *p* = 0.408
LTI (kg)	***r* = 0.377** ***p* < 0.001**	***r* = 0.419** ***p* < 0.001**	*r* = 0.001 *p* = 0.998	*r* = −0.212 *p* = 0.06
LTM (kg/ m^2^)	***r* = 0.296** ***p* = 0.007**	***r* = 0.238** ***p* = 0.002**	*r* = 0.110 *p* = 0.327	*r* = 0.029 *p* = 0.803
DPI (g/kg/d)	*r* = −0.144 *p* = 0.161	*r* = −0.131 *p* = 0.204	*r* = −0.111 *p* = 0.280	*r* = 0.164 *p* = 0.114

CRP, C-reactive protein; BMI, body mass index; ST, skinfold thickness; MAC, mid-arm circumference; LTM, lean tissue mass; LTI, lean tissue index; DPI, dietary protein intake. The bolding values mean statistically significant correlations between the variables.

### 3.5 Associations of the relative abundance of different gut flora with decrease in LTM

Decreased LTM is one of the characteristics of PEW that is associated with the prognosis of patients with maintenance hemodialysis. Therefore, we used binary logistic regression analysis to determine whether abundance of any of the identified gut microbiota taxa was associated with a decrease in LTM. As shown in Table 3, increase in the abundance of Actinobacteria (at the class level) was associated with a lower likelihood of decreased LTM 1 year later [for >5% decrease: unadjusted OR = 0.72 (0.55–0.95), adjusted OR = 0.73 (0.55–0.97), *p* < 0.05; for >10% decrease: unadjusted OR = 0.70 (0.51–0.97), adjusted OR = 0.73 (0.53–0.99), *p* < 0.05]. Similarly, increase in the abundance of Bifidobacteriaceae at the family level was also related to a lower likelihood of decreased LTM after 1 year [for >5% decrease: unadjusted OR = 0.65 (0.46–0.92), adjusted OR = 0.66 (0.46–0.95), *p* < 0.05; for >10% decrease: unadjusted OR = 0.56 (0.35–0.92), adjusted OR = 0.59 (0.37–0.94), *p* < 0.05]. On the other hand, increase in the abundance of *Pseudocitrobacter* at the genus level or *C. clostridioforme* at the species level does not seem to be related to decrease in LTM (*p* > 0.05).

**Table 3:** Odds ratio and 95% confidence intervals for decrease in lean tissue mass according to the abundances of gut microbiota.

Taxa	Model	LTM decrease >5%	*p*-value	LTM decrease >10%	*p*-value
c__Actinobacteria	A	**0.72 (0.55–0.95)**	**0.019**	**0.70 (0.51–0.97)**	**0.032**
	B	**0.73 (0.55–0.97)**	**0.028**	**0.73 (0.53–0.99)**	**0.044**
f__Bifidobacteriaceae	A	**0.65 (0.46–0.92)**	**0.015**	**0.56 (0.35–0.92)**	**0.022**
	B	**0.66 (0.46–0.95)**	**0.026**	**0.59 (0.37–0.94)**	**0.025**
g__Pseudocitrobacter	A	1.14 (0.75–1.75)	0.54	1.11 (0.82–1.49)	0.50
	B	1.11 (0.76–1.63)	0.58	1.09 (0.82–1.45)	0.57
s__Clostridium_clostridioforme	A	0.94 (0.73–1.22)	0.66	1.02 (0.79–1.32)	0.90
	B	0.98 (0.71–1.34)	0.89	1.02 (0.74–1.40)	0.91

A: unadjusted, B: adjusted for age, sex, hemoglobin, PTH, ferritin, CRP, dialysis duration and kt/v LTM, lean tissue mass. The bolding values mean statistically significant correlations between the dependent variables (decrease in LTM) and the independent variables (the relative abundance of gut microbiota).

**Figure 5:**
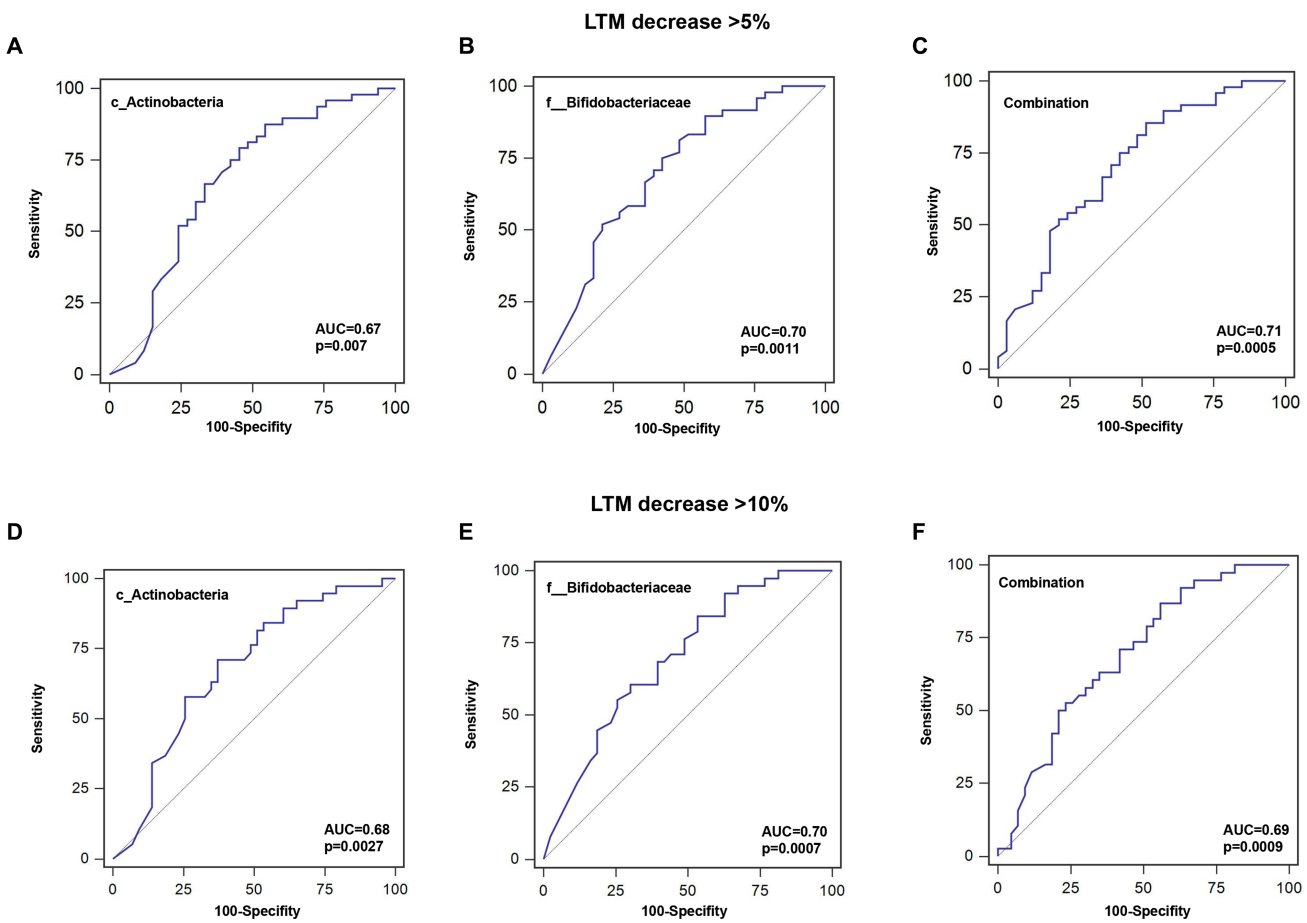
ROC curves for predicting the probability of lean tissue mass decrease. **(A–C)** Sensitivity, specificity, and AUC for c_Actinobacteria, f__Bifidobacteriaceae, or their combination for a >5% decrease in LTM. **(D–F)** Sensitivity, specificity, and AUC for c_Actinobacteria, f__Bifidobacteriaceae, or their combination for a >10% decrease in LTM.

To further investigate the predictive value of the abundance of identified gut microbiota taxa for decrease in LTM, the AUC area was calculated. As shown in Figure 5 and Table 4, the sensitivity of c_Actinobacteria for predicting >5 and >10% decrease in LTM was 79.2% and 71.1%, respectively, while the specificity was 54.6% and 62.8%, respectively, with corresponding AUC values of 0.676 and 0.681. The sensitivity of f_ Bifidobacteriaceae in predicting >5% and >10% decrease in LTM was 81.3% and 84.2%, respectively, while the specificity was 51.5% and 46.5%, respectively, with corresponding AUC values of 0.700 and 0.697. The combined use of c_Actinobacteria and f_Bifidobacteriaceae slightly increased the accuracy of predicting >5% decrease in LTM, but not for predicting >10% decrease.

**Table 4:** Evaluation of the efficacy of abundances of gut microbiota for predicting decrease in LTM.

Taxa	AUC (95% CI)	Cutoff Value	Sensitivity (%)	Specificity (%)	Accuracy (%)	*p*-value
**LTM decrease >5%**						
c__Actinobacteria	0.676 (0.563–0.776)	0.49	79.2 (38/48)	54.6 (18/33)	69.1 (56/81)	0.007
f__Bifidobacteriaceae	0.700 (0.588–0.797)	0.48	81.3 (39/48)	51.5 (17/33)	69.1 (56/81)	0.0011
Combination	0.708 (0.596–0.804)	/	85.4 (41/48)	48.5 (16/33)	70.4 (57/81)	0.0005
**LTM decrease >10%**						
c__Actinobacteria	0.681 (0.568–0.780)	0.36	71.1 (27/38)	62.8 (27/43)	66.7 (54/81)	0.003
f__Bifidobacteriaceae	0.697 (0.585–0.794)	0.48	84.2 (32/38)	46.5 (20/43)	64.2 (52/81)	0.0007
Combination	0.693 (0.581–0.791)	/	86.9 (33/38)	44.2 (19/43)	64.2 (52/81)	0.0009

### 3.6 Associations of the abundance of different gut microbiota with the development of PEW

**Figure 6:**
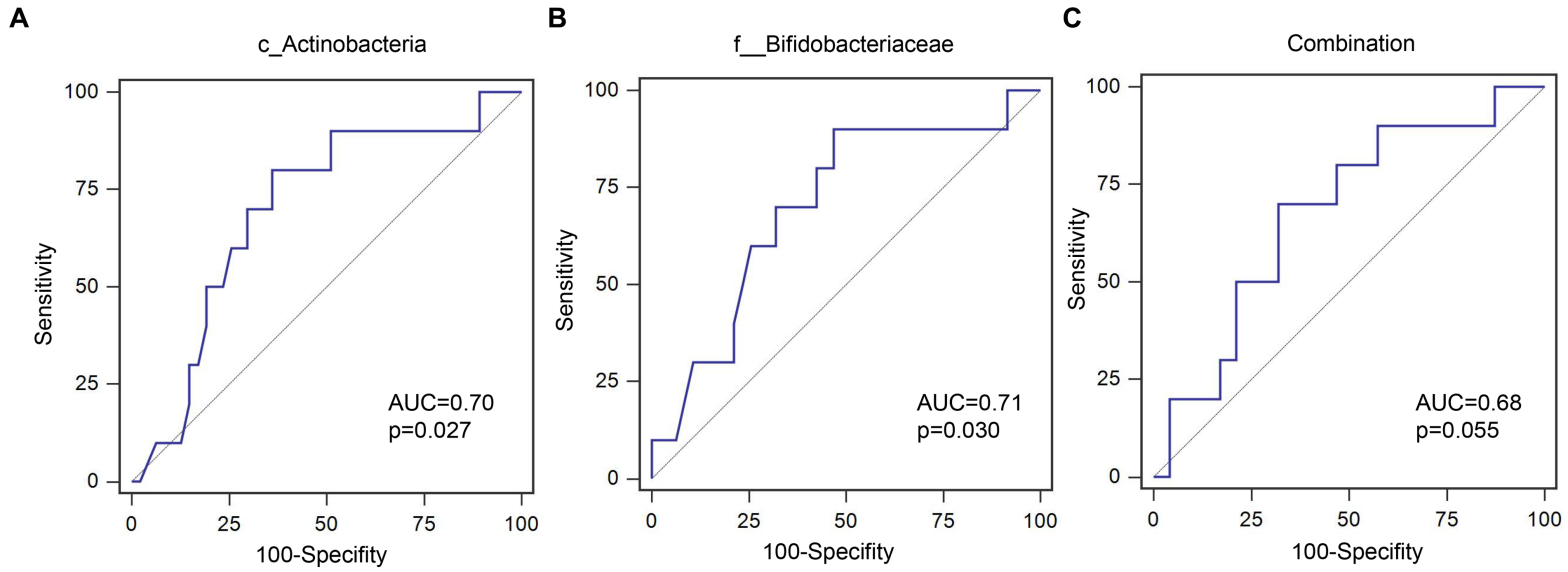
ROC curves for predicating the probability of PEW development. **(A)** c_Actinobacteria; **(B)** f__Bifidobacteriaceae; **(C)** Combination of c_Actinobacteria and f__Bifidobacteriaceae.

Out of 60 patients who were not diagnosed with PEW at baseline, 57 were followed up for 1 year, of whom 10 developed PEW over the 1 year period. Using AUC analysis (shown Figure 6), we found that the relative abundance of c_Actinobacteria and f_Bifidobacteriaceae at baseline may help to predict the development of PEW (c_Actinobacteria: AUC = 0.70, *p* = 0.027; f_Bifidobacteriaceae: AUC = 0.71, *p* = 0.03). Group analysis using binary logistic regression analysis showed that a decreased likelihood of PEW development occurred in the patients with higher relative abundance of c_Actinobacteria [adjusted OR = 0.15 (0.029–0.82), *p* = 0.028] or f_Bifidobacteriaceae [adjusted OR = 0.092 (0.011–0.79), *p* = 0.030] compared with the lower relative abundance group (shown in Table 5). Taking into consideration the correlation of the abundance of these taxa with albumin concentrations and LTM, it appears that a decrease in their abundance may contribute to the development of PEW.

**Table 5:** Odds ratio and 95% confidence intervals for PEW development according to relative abundances of gut microbiota.

Taxa	Model	Ctrl	PEW	*p*-value
c__Actinobacteria (category)	A	1.00	0.14 (0.027–0.75)	0.021
	B	1.00	0.15 (0.029–0.82)	0.028
c__Actinobacteria (continuous)	A	/	0.77 (0.49–1.21)	0.26
	B	/	0.72 (0.40–1.30)	0.27
f__Bifidobacteriaceae (category)	A	1.00	0.082 (0.010–0.70)	0.023
	B	1.00	0.092 (0.011–0.79)	0.030
f__Bifidobacteriaceae (continuous)	A	/	0.79 (0.50–1.26)	0.33
	B	/	0.72 (0.39–1.33)	0.29

Odds ratios (95% CI) for comparison of the relative abundance of each bacterium taxon (according to whether it is above or below the cutoff values from ROC curve) for prediction of PEW development. Category variables means assignment to higher or lower relative abundance group according to cutoff values from ROC analysis (0.36 for c__Actinobacteria and 0.39 for f__Bifidobacteriaceae). A: unadjusted, B: adjusted for age, sex, hemoglobin, PTH, ferritin, CRP, dialysis duration, and kt/v Ctrl refers to patients with taxon abundance below the cutoff value.

## 4 Discussion

The present observational longitudinal study investigated the potential role of microbial taxa in muscle mass reduction and PEW occurrence in patients on hemodialysis through an examination of their gut microflora composition at baseline and over a 1 year period following hemodialysis. After the 1 year follow-up, we analyzed the associations between the abundance of various microbial taxa and variations in LTM and the development of PEW. We found that the relative abundances of Actinobacteria at the class level, Bifidobacteriales at the order level, and Bifidobacteriaceae at the family level were significantly lower in the PEW group. Further, abundance of Actinobacteria and Bifidobacteriaceae correlated positively with variations of serum albumin levels, LTM, and LTI. Relatively low abundance of Actinobacteria and Bifidobacteriaceae may act as predictors of the likelihood of LTM decrease and PEW occurrence. These findings indicate that a decrease in the abundance of Actinobacteria and Bifidobacteriaceae in patients on hemodialysis may be involved in the development of PEW.

Substantial evidence from human studies has shown that CKD is associated with gut microbiota dysbiosis. Compared to healthy individuals, CKD patients have a lower relative abundance of bacteria from the phyla Firmicutes and Actinobacteria (19). Further, the Enterococcus and Clostridium families are also more abundant in patients with CKD, while the Prevotella, Coprococcus, Megamonas, Sutterella, Enterobacter, Acidaminococcus, Dorea, and Roseburia families are enriched in healthy individuals. In another study, patients with ESRD were found to have a greater abundance of bacterial families that produce urease, uricase, and other enzymes capable of forming uremic toxins such as indole and p-cresol. The influx of urea into the intestinal lumen as a result of ESRD could lead to an overgrowth of bacteria that produce urease and affect the growth of commensal bacteria (20). Recently two cross-sectional studies indicated a marked reduction of bacteria producing butyrate or butyric-acid in hemodialysis patients with PEW (16, 17). All the studies including our study demonstrated disruption of intestinal flora in PEW patients. One study found that *Bifidobacterium* at genus level was slightly increased in PEW patients, which was different from our findings. Possible reasons for this difference are that in that study patients were evaluated by SGA scores instead of PEW diagnosis criteria and severe PEW patients were excluded in that study. Our observational longitudinal study further explored in which way gut microbiota dysbiosis participated in the development of PEW.

Consistent inflammation has been recognized as one of the important components of PEW development. Thus, gut microbiota disorders may probably be involved in the consistent inflammation detected in PEW (21). In this study, we found that the relative abundance of *C. clostridioforme* was significantly increased in the PEW group compared with the non-PEW group. Clostridials are the main producers of butyrate, which is necessary for maintaining the integrity of the colonic mucosa and promoting anti-inflammatory pathways. Accordingly, depletion of butyrate-producing Clostridia from the gut microbiota has been found to promote the growth of facultative anaerobes in the mouse intestine (22). In contrast to these findings, another report showed that *C. clostridioforme* could stimulate consistent release of pro-inflammation cytokines from peripheral blood mononuclear cells (23). In the present study, we found that the relative abundance of *C. clostridioforme* correlated positively with variations in serum albumin levels. Serum albumin is closely associated with protein metabolism in skeletal muscle, which is the largest protein storage organ in the body, and hypoproteinemia is a strong predictor of mortality in patients with ESRD. Therefore, given the inconsistency between these findings, whether the increase in the abundance of Clostridials was beneficial to patients with PEW requires further investigation.

According to previous studies, Bifidobacteriaceae may affect skeletal muscle mass through the action of its metabolites, such as acetate propionate and butyrate, which play a central role in regulating immune and metabolic homeostasis (24). In adolescents with obesity, the relative abundances of Actinobacteria and Bifidobacteria were positively associated with serum short-chain fatty acid concentrations, which could enhance adaptive fat oxidation, decrease markers of protein catabolism, and modulate inflammatory responses (25). Bifidobacteriaceae also promotes the absorption and utilization of vitamin D and minerals, which are essential for skeletal muscle metabolism (26). Further, a recent study revealed that the Bifidobacteriaceae genome is rich in genes encoding for proteins involved in carbohydrate and amino acid transport and metabolism, which possibly also affect protein synthesis, nutrient utilization, and skeletal muscle mass (27). Accordingly, in the present study, it was shown that the relative abundances of Actinobacteria and Bifidobacteriaceae may help to predict variations in skeletal muscle mass, based on the results of ROC curve analysis. Thus, variations in the abundance of Actinobacteria and Bifidobacteriaceae may be involved in the decrease in skeletal muscle mass. In addition, the relative abundances of Actinobacteria and Bifidobacteriaceae may also help to predict the development of PEW, according to the findings from the 1 year follow-up of patients who were not diagnosed with PEW at the baseline. We may infer from the logistic regression model that decrease in the abundance of Actinobacteria and Bifidobacteriaceae may have potentially contributed to the development of PEW, although the results were not significant on account of the relatively small study population.

Decreased muscle mass is one of the characteristics of PEW. Several studies indicated that increase in Bifidobacteriaceae abundance may improve the mass of the gastrocnemius and tibialis muscles, enhance muscle functions such as grip strength, and ameliorate muscle atrophy both in aged mice and super-elderly patients (28, 29). Further, low abundance of Bifidobacteriaceae may be a potential marker of sarcopenia in elderly woman (30). However, in critically ill patients who were orally administered *Bifidobacterium* for 14 days, protein homeostasis and muscle mass did not show significant improvement (31). On the other hand, in anorectic patients, weight gain is associated with an increase in the abundance of Bifidobacteria (32). Modifications in gut microbiota composition in the starvation state may be the result of an adaptive response to optimizing food transformation in individuals on low-calorie diets. However, the pathogenesis of PEW is considerably different from that of anorexia. It has been noted that highly processed food typically lower in fiber, antioxidants and prebiotics may lead to an imbalance in the gut microbiota (33). Up till now, there are few reports about the associations between *Bifidobacterium* and PEW in patients on maintenance hemodialysis. A clinical trial in patients with diabetes on hemodialysis demonstrated that administration of a probiotic mixture including *Bifidobacterium bifidum* for 12 weeks significantly improved the nutritional status of patients based on the SGA score (34). In addition, a randomized double-blind and controlled trial recently demonstrated that administration of fortified synbiotic dessert effectively reduced the severity of malnutrition in hemodialysis patients (35). Our longitudinal data demonstrate, for the first time, that decrease in the relative abundance of Actinobacteria and Bifidobacteriaceae may be associated with skeletal muscle mass decrease and PEW development in hemodialysis patients. Thus, Actinobacteria and Bifidobacteriaceae may be potential predictors of variations in skeletal muscle mass and PEW status during the follow-up period after hemodialysis.

There are several limitations to this study. First, the population was relatively small. Second, we did not record the dietary pattern of individual patients. Difference in dietary patterns may affect the components of gut microbiota. Third, we did not record the fiber intake from the dietary which may be associated with malnutrition in the hemodialysis patients. Fourth, no experiments were conducted to verify the involvement of certain microbiota in skeletal muscle mass decrease and PEW development. Future research is warranted to clarify these issues.

## 5 Conclusion

The present analysis of gut microbiota composition in patients on hemodialysis who develop PEW revealed that a decrease in the abundance of Actinobacteria at the class level and Bifidobacteriaceae at the family level may be associated with skeletal muscle mass decrease and PEW development. Thus, assessment of the intestinal flora in patients on hemodialysis may help predict the possibility of PEW development, and this could facilitate timely intervention and, perhaps, improve their prognosis. Further studies are required to confirm the precise role of *Bifidobacterium* in skeletal muscle metabolism and PEW development.

## Data availability statement

The datasets presented in this study can be found in online repositories. The names of the repository/repositories and accession number(s) can be found in the article/supplementary material.

## Ethics statement

The studies involving humans were approved by Ethics Committee of the Shanghai Ninth People’s Hospital, School of Medicine, Shanghai Jiaotong University (SH9H-2019-T322-2). The studies were conducted in accordance with the local legislation and institutional requirements. The participants provided their written informed consent to participate in this study. Written informed consent was obtained from the individual(s) for the publication of any potentially identifiable images or data included in this article.

## Author contributions

XB: Formal analysis, Investigation, Methodology, Writing – original draft, Writing – review & editing, Funding acquisition. YL: Data curation, Writing – review & editing, Writing – original draft. LY: Data curation, Writing – review & editing. LL: Data curation, Writing – review & editing. JL: Methodology, Resources, Writing – review & editing. CH: Funding acquisition, Writing – review & editing. WD: Funding acquisition, Project administration, Writing – review & editing.
